# Downregulation of 15-hydroxyprostaglandin dehydrogenase during acquired tamoxifen resistance and association with poor prognosis in ERα-positive breast cancer

**DOI:** 10.37349/etat.2020.00021

**Published:** 2020-10-30

**Authors:** Milene Volpato, Michele Cummings, Abeer M. Shaaban, Balkees Abderrahman, Mark A. Hull, Philipp Y. Maximov, Bradley M. Broom, Reiner Hoppe, Ping Fan, Hiltrud Brauch, V. Craig Jordan, Valerie Speirs

**Affiliations:** 1 Leeds Institute of Medical Research, University of Leeds, St James’s University Hospital, LS9 7TF Leeds, UK; 2 Institute of Cancer and Genomic Sciences, University of Birmingham, B15 2TT Birmingham, UK; 3 Department of Breast Medical Oncology, UT MD Anderson Cancer Center, Houston, TX 77030, USA; 4 Department of Bioinformatics and Computational Biology, UT MD Anderson Cancer Center, Houston, TX 77030, USA; 5 Dr. Margarete Fischer-Bosch Institute of Clinical Pharmacology and University of Tübingen, Auerbachstr. 112, D-70376 Stuttgart, Germany; 6 Germany iFIT Cluster of Excellence, University of Tübingen, Auerbachstr. 112, D-70376 Stuttgart, Germany; 7 German Cancer Consortium (DKTK) and German Cancer Research Center (DKFZ), Im Neuenheimer Feld 280, 69120 Heidelberg, Germany; 8 Institute of Medical Sciences, University of Aberdeen, Foresterhill, AB25 2ZD Aberdeen, UK; University of Edinburgh, UK

**Keywords:** Breast cancer, endocrine resistance, 15-hydroxyprostaglandin dehydrogenase, immunohistochemistry, survival

## Abstract

**Aim:**

Tamoxifen (TAM) resistance remains a clinical issue in breast cancer. The authors previously reported that 15-hydroxyprostaglandin dehydrogenase (*HPGD*) was significantly downregulated in tamoxifen-resistant (TAMr) breast cancer cell lines. Here, the authors investigated the relationship between HPGD expression, TAM resistance and prediction of outcome in breast cancer.

**Methods:**

*HPGD* overexpression and silencing studies were performed in isogenic TAMr and parental human breast cancer cell lines to establish the impact of HPGD expression on TAM resistance. HPGD expression and clinical outcome relationships were explored using immunohistochemistry and *in silico* analysis.

**Results:**

Restoration of *HPGD* expression and activity sensitised TAMr MCF-7 cells to TAM and 17β-oestradiol, whilst *HPGD* silencing in parental MCF-7 cells reduced TAM sensitivity. TAMr cells released more prostaglandin E_2_ (PGE_2_) than controls, which was reduced in TAMr cells stably transfected with *HPGD*. Exogenous PGE_2_ signalled through the EP4 receptor to reduce breast cancer cell sensitivity to TAM. Decreased HPGD expression was associated with decreased overall survival in ERα-positive breast cancer patients.

**Conclusions:**

HPGD downregulation in breast cancer is associated with reduced response to TAM therapy via PGE_2_-EP4 signalling and decreases patient survival. The data offer a potential target to develop combination therapies that may overcome acquired tamoxifen resistance.

## Introduction

Due to its ability to bind to and modulate oestrogen receptor alpha (ERα) activity, Tamoxifen (TAM) was the first targeted therapy for breast cancer [1], with its widespread use in the clinic now extending to four decades. While aromatase inhibitors are now the preferred first line endocrine treatment in the post-menopausal setting [2], TAM is favoured in pre-menopausal breast cancer patients [3]. However, approximately 70% of patients initially respond to TAM, but most eventually acquire resistance [2]. Acquisition of TAM-resistance (TAMr) continues to be a major limitation for long-term management of breast cancer patients. However, the mechanism(s) responsible for TAM insensitivity is not yet fully understood.

In the last three decades, various groups have developed cell line models in order to help unravel the mechanistic basis of TAMr. Most of these have been achieved through long term culture of the hormone-sensitive MCF-7 human breast cancer cells in sub-lethal doses of TAM, during which resistant sub-clones develop after 3–6 months [4–8]. Using two independently-derived ER positive MCF-7 human breast cancer cell models of acquired TAMr developed in our laboratory [9], an Affymetrix^®^ microarray study showed that 131 genes were upregulated and 156 were downregulated by at least 3-fold, in TAMr MCF-7 cells relative to isogenic control cells [10]. One of the genes that were downregulated was 15-hydroxyprostaglandin dehydrogenase (HPGD). HPGD (EC 1.1.1.141) is a member of the short-chain alcohol (OH) dehydrogenase family and is the key NAD^+^-dependent enzyme responsible for the biological inactivation of prostaglandins, including prostaglandin E_2_ (PGE_2_), which is synthesized via the cyclooxygenase (COX) pathway.

Downregulation of HPGD has been shown in several malignancies including lung, colon, bladder, endometrial, and gastric cancer and has been shown to have a tumour suppressor roles in some settings [11–16]. Studies in breast cancer are contradictory; higher *HPGD* expression has been reported in ERα-positive MCF-7 cell with reduced expression in ERα-negative MDA-MB-231 cells, where up-regulation was observed following treatment with demethylating agents [17]. In clinically more aggressive primary breast cancers, particularly those with a triple negative phenotype, and in breast cancer metastases, HPGD is overexpressed, with high HPGD expression associated with poor prognosis and reduced survival [18]. In the same study, *HPGD* silencing induced a mesenchymal-epithelial transition resulting in a less migratory phenotype. More recently, data mining from publicly available breast cancer gene expression datasets from the Cancer Genome Atlas (TCGA, https://www.cancer.gov/about-nci/organization/ccg/research/structural-genomics/tcga) and Oncomine (https://www.oncomine.org/), revealed decreased expression of *HPGD* mRNA in breast cancer samples compared with normal, healthy breast tissue [19]. Furthermore, HPGD expression was lower still in more aggressive basal/triple negative and HER2-positive breast cancers. Conversely, in rare apocrine breast carcinomas, HPGD was highly expressed [20]. Application of a triclustering algorithm, δ-TRIMAX to microarray datasets characterized *HPGD* as one of ten so-called hub-genes associated with breast cancer [21], lending support for a role in breast carcinogenesis.

Building on our previous data [10], the aim of this study was to investigate a causal link between *HPGD* expression and function and TAMr, as well as explore the relationship between HPGD expression in breast cancer samples and clinical outcomes. The effects of HPGD expression on outcome were examined through *in silico* analysis and by immunohistochemistry in a breast cancer cohort with long term follow up, including breast cancer outcomes during adjuvant TAM.

## Materials and methods

### Cell lines and culture

The two stable TAMr MCF-7 human breast cancer cell lines have been described previously [9, 10]. All MCF-7 isogenic lines were cultured in phenol-red-free RPMI 1640 containing L-glutamine (Invitrogen, UK) supplemented with 5% charcoal-stripped steroid-depleted foetal calf serum (FCS; Harlan SeraLab, UK), 100 U/mL penicillin and 100 U/mL streptomycin and 100 nM 4-hydroxytamoxifen (TAM; Sigma-Aldrich, UK) for 12–24 months. Parental cells [termed wild type (WT) MCF-7] were cultured in the same medium, but with 0.01% (v/v) ethanol vehicle. Experiments were conducted in phenol-red-free RPMI 1640 supplemented with 5% charcoal-stripped steroid-depleted FCS + TAM (0.1–100 nM) or 17β-oestradiol (E2; 0.001-1 nM). Bi-monthly mycoplasma checks were consistently negative and annual short tandem repeat (STR) profiling confirmed cell provenance, both carried out as a service in Leeds. To minimize genetic drift, cell stocks were frozen at low passage and experimental cultures replaced from these stocks every 3–6 months. We also studied LCC1, -2 and -9 breast carcinoma cells, which were derived originally in the Clarke laboratory at Georgetown, Washington DC and cultured as described [7, 8, 22]. LCC1 cells are estrogen-independent and -responsive TAM-sensitive breast cancer cells, which are derived from an estrogen-independent variant of MCF-7 cells (MCF7/MIII) through *in vivo* selection in oophorectomized nude mice (with circulating estrogen levels similar to a postmenopausal woman), and subsequently re-cultured *in vitro* to become a stable cell line. LCC2 cells are stable, ER-positive, estrogen-independent, TAM-resistant, and respond to fulvestrant [7]. They were derived from the selection of LCC1 for TAM resistance *in vitro*. LCC9 cells exhibits cross resistance to TAM and are unresponsive to fulvestrant [7]. They were derived from the selection of LCC1 for fulvestrant resistance *in vitro*. MCF7:5C and MCF-7:WS8 are oestrogen-independent and dependent, respectively, ER-positive, progesterone receptor (PR)-negative, and TAM-resistant breast cancer cells [23]. They were derived from WT MCF-7 cells following long-term estrogen deprivation (LTED). The main characteristics of the cell lines used are detailed in Table 1.

**Table 1.** Characteristics of TAM-resistant breast cancer cell lines derived from MCF-7

**Parental cell line**	**TAM resistant derivative**	
	**Cell line**	**Method of induction of TAM resistance**
WT MCF-7 (oestrogen-dependent, TAM-sensitive)	TAMr 1	Chronic TAM exposure (12 months)
	TAMr 2	Chronic TAM exposure (21 months)
	MCF-7-5C	Long-term oestrogen deprivation
	MCF-7-WS8	Long-term oestrogen deprivation
MCF-7/LCC1 (oestrogen-independent, endocrine therapy responsive)	LCC2	Stepwise selection against TAM
	LCC9	Stepwise selection against fulvestrant

### Microarray analysis of LCC1, -2 and -9

Cells were seeded at 300, 000 cells per well in 6-well plates (Corning, UK). They were treated the next day with 1 nM E2 for the indicated durations. All experiments were performed in triplicate. Subsequently, cells were harvested in TRIzol (Life Technologies, UK) and total RNA was isolated using the Qiagen RNeasy RNA purification kit (Qiagen, UK). Isolated RNA (1 µg) was then processed on the Affymetrix Clariom S microarray platform (Affymetrix, UK) and used to determine the significance of differential expression of *HPGD* at basal levels.

### 3-(4,5-dimethylthiazol-2-yl)-2,5-diphenyltetrazolium bromide (MTT) assay

Metabolic activity was used as a surrogate for cell proliferation, using the 3-(4,5-dimethylthiazol-2-yl)-2,5-diphenyltetrazolium bromide (MTT) assay [9]. Cells were seeded into 96-well plates. After overnight attachment, TAM (0.1-100 nM), E2 (0.01–1 µM), PGE_2_ (1 µM), butaprost or PGE_1_-OH (both 0.1–1 µM) or ethanol vehicle was added. At 96 h post-treatment, cells were incubated with 1 mM MTT for 4 h. The purple formazan product was solubilised in propan-1-ol and read at 570 nm using an Opsys MR^TM^ 15 (Dynex Technologies, UK). Chemicals were purchased from Sigma-Aldrich (UK) or Cayman Chemical (USA).

### Western blot analysis

This was conducted as described previously [24], except that HPGD was detected using a rabbit polyclonal antibody (catalogue number NB200-179, Novus Biological, UK, 1:500). Bands were then visualised with Supersignal West pico ECL reagent (ThermoFisher, UK).

### Real-time polymerase chain reaction (RT-PCR)

Total RNA was extracted, and reverse transcribed as described previously [24]. Real-time polymerase chain reaction (RT-PCR) for *HPGD* or prostaglandin E_2_ receptor 4 (*EP4*) mRNA was performed using a SYBR Green-based assay (ThermoFisher, UK) on an ABI 7700 sequence detection system using primers for *HPGD* (forward, 5’-TAGTTGGATTCACACGCTCAGC-3’; reverse, 5’-AAAGCCTGGACAAATGGCAT-3’) and *EP4* (forward, 5’-TCTTACTCATTGCCACCTCCCT-3’; reverse, 5’-CTTGGCTGATATAACTGGTTGACG-3’). The ribosomal protein gene *RPLPO* was used as the reference gene (forward, 5’-GAAACTCTGCATTCTCGCTTCC-3’; reverse, 5’-GATGCAACAGTTGGGTGCCA-3’). Levels of HPGD or EP4 transcripts were quantified using the 2-ΔCt method [25].

### *HPGD* transfections

A stab culture of human *HPGD* cDNA IMAGE clone ID 3638799 was obtained from MRC and *HPGD* was PCR-amplified from this using the following primers: forward, 5′-CCGGGATCCTGCACCATGCACGTGAAC-3′; reverse, 5′-CCCCAAGCTTTCATTGGGTTTTTGCTTG-3′. Products were cloned into pcDNA3.1(-)myc/his (gift from Dr Thomas Hughes, University of Leeds). Vectors containing *HPGD* or empty vector controls were transfected into TAMr cells using Lipofectamine 2000 (ThermoFisher, UK). Stable clones were selected and maintained in G418 (ThermoFisher, UK)-containing medium (250 µg/mL).

### PGE_2_ ELISA and HPGD enzyme activity assay

Both assays were performed using previously protocols published [26]. For PGE_2_, serum-free conditioned medium was collected from cells after overnight incubation, and PGE_2_ levels were measured using a competitive immunoassay (Amersham Biosciences, UK), and normalised to total cellular protein content as measured by Bradford assay. HPGD activity was measured in lysates of WT MCF-7 and TAMr cells by assessing the transfer of tritium from ^3^H-PGE_2_ to glutamate, catalysed by HPGD and glutamate dehydrogenase [27].

### EP4 and HPGD knockdown

Small-interfering (si)-RNAs targeting *EP4* or *HPGD* (ThermoFisher Scientific, UK) were reverse transfected into cells using Lipofectamine 2000 according to the manufacturer’s instructions. Briefly, MCF-7 cells were mixed with media containing small-interfering RNA (siRNA)/lipid (final siRNA concentration 10 nM) and seeded into 96 well plates at 0.4 x 10^4^ cells/well and allowed to establish overnight before incubation with drug or vehicle control. Response to treatment was assessed by MTT assay. *EP4* or *HPGD* knockdown was assessed by real-time quantitative polymerase chain reaction (qRT-PCR) as described previously [28].

### Immunohistochemistry

Following ethical approval (06/Q1206/180) we investigated HPGD expression in 350 primary invasive breast cancers, all of which, had been surgically resected and received adjuvant TAM, and were represented on tissue microarrays (TMAs) [24, 29]. Out of the 350 cases, 108 cases experienced a relapse (TAMr) and 242 cases did not [tamoxifen-sensitive (TAMs)]. Mean follow-up was 80 months [range 1–229, standard deviation (SD) 44.2]. Tumour staining was achieved using previously described methods [24, 26].

### *In silico* transcriptomic analysis

The relationship between *HPGD* expression and cancer outcomes was analyzed using Kaplan-Meier Plotter (KMplot, https://kmplot.com/analysis/) [30]. Using the 2017 release of the database, a cohort of ERα-positive breast tumours, previously treated with TAM only and no adjuvant chemotherapy, was analyzed and the association between *HPGD* expression and overall survival (OS). Patients were dichotomized as high or low HPGD expression using lower tertile as a cut-off. Multivariate analysis was done using in-built software with Ki67, ER and HER2 as covariates.

### *In silico* analyses for the identification of miRNAs targeting *HPGD*

We applied miRWalk version 3.0 [31] and TargetScan version 7.2 [32] to a previously described Affymetrix miRNA dataset of endocrine sensitive MCF-7:WS8 and resistant MCF-7:5C cells [33] in order to identify miRNA-target interactions sites for major *HPGD* transcripts using default settings. OncoLnc (http://www.oncolnc.org/) was used to retrieve *HPGD* and miR-3200-3p expression data from matching breast cancer cases in the TCGA database [34]. Kaplan-Meier Plotter [30] was used to investigate the significance of miR-3200-3p on survival in ERα+ breast cancer treated with any endocrine therapy (not limited to TAM). Cases were dichotomised as high or low miR-3200-3p expression using the median expression value as a cut-off.

### Statistical analysis

For *in vitro* analyses, one-way ANOVA was performed. The log rank test was used to compare patient survival in the primary breast cancer cohort. Analyses were performed using GraphPad Prism version 7.03 (GraphPad Software, La Jolla California, USA). For Affymetrix analyses, ANOVA *P*-values were calculated using the Affymetrix Transcriptome Analysis Console (Affymetrix). The time course plots were plotted as fold-change with the baseline value set at 1.

## Results

### HPGD is downregulated in TAMr MCF-7^MMU2^ cells

**Figure 1.**
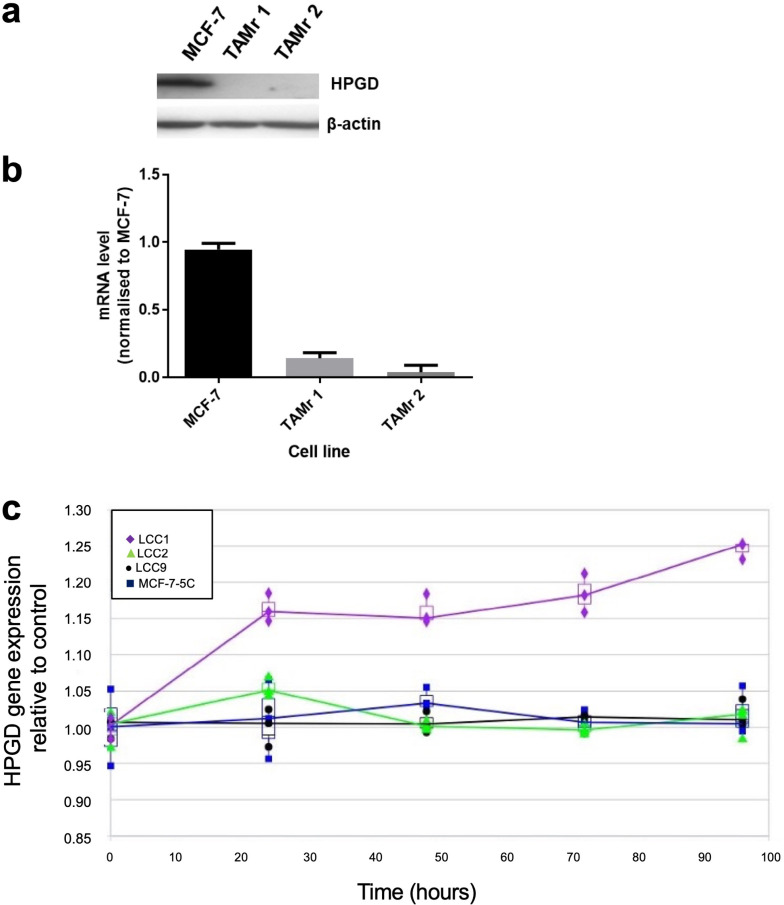
HPGD is downregulated in TAMr derivatives of MCF-7 cells. Western blot (a) showing HPGD protein expression in WT MCF-7 and its loss in two, independently derived, TAMr MCF-7 cell lines, TAMr 1 and TAMr 2; qRT-PCR (b) shows relative expression of *HPGD* mRNA in these cell lines. Expression was determined using *RPLP0* as the reference gene and further normalized to baseline at time 0 h (± SD); in (c), expression of *HPGD* mRNA in LCC1 cells (TAMs) *versus* LCC2 cells (TAMr), LCC9 (TAMr and fulvestrant resistant), and MCF7:5C (TAMr) shows an upregulation over time in LCC1 cells only, with the resistant variants remaining largely unchanged during treatment with 1 nM E2

We demonstrated that HPGD was downregulated in two independent TAMr MCF-7 cell lines, which had been cultured continuously in 100 nM TAM for 12 (TAMr 1) and 21 (TAMr 2) months [10], respectively by Western blot (Figure 1a) and real time PCR (Figure 1b) compared with parental MCF-7 cells. Microarray analysis of the independent LCC cell line series and MCF7:5C cells showed that only the oestrogen-independent but oestrogen-responsive TAMs cell line LCC1 showed increased expression of HPGD in response to E2 over time (Figure 1c).

### Effect of HPGD overexpression in MCF-7 TAMr 2 cells

**Figure 2.**
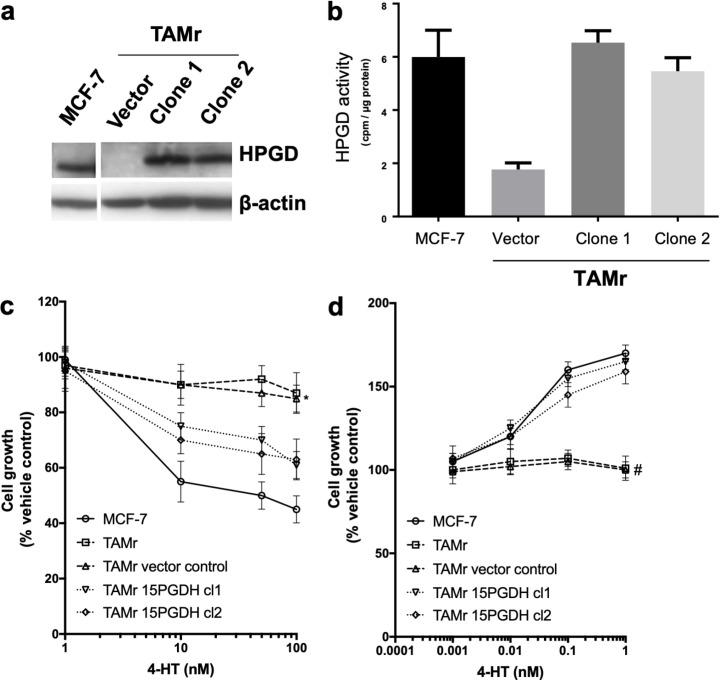
Effect of overexpressing *HPGD* in MCF-7 TAMr 2 cells. Western blot (a) showing HPGD expression in stably transfected clones of MCF-7 TAMr 2 cells compared to empty vector control and WT MCF-7; in (b), HPGD enzyme activity (cpm/μg protein) is restored to levels similar to WT MCF-7 following *HPGD*-transfection, sensitising cells to inhibitory effects of TAM (* *P* < 0.05 *vs.* MCF-7, Figure 2c) and restoring E2 sensitivity (# *P* < 0.004 *vs.* MCF-7, Figure 2d). Data are representative of triplicate experiments (± SEM)

We then explored the effects of introduction of *HPGD* into MCF-7 TAMr 2 cells. Successful expression of HPGD protein following stable transfection was confirmed by Western blotting (Figure 2a). This restored activity of HPGD in each of the transfected cell lines to levels like WT MCF-7 (Figure 2a and b). This also sensitized these cells to the inhibitory effects of TAM to approximately 60% of the parental MCF-7 cell response (Figure 2c), whilst restoring sensitivity to E2 almost completely (Figure 2d).

### Modulating the PGE_2_ axis influences cellular response to TAM

**Figure 3.**
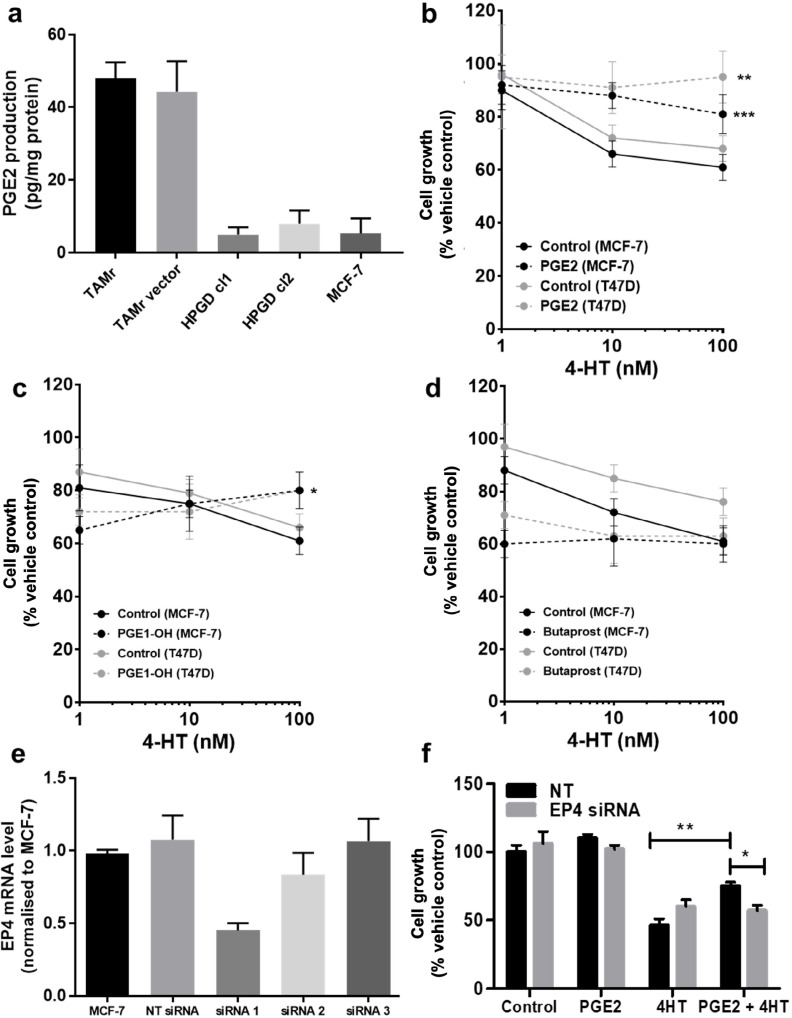
Modulating the PGE_2_ axis influences cellular response to TAM. (a) Overexpression of *HPGD* reduced PGE_2_ production in MCF-7 TAMr 2 cells to similar levels to basal production in WT MCF-7 cells; (b) exogenous PGE_2_ (1 μM, broken lines) reduced sensitivity of WT MCF-7 and T47D human breast cancer cells to TAM; (c) PGE_1_-OH mimicked the effects PGE_2_ in decreasing TAM sensitivity in WT MCF-7 and T47D cells, albeit to a lesser extent; (d) butaprost was ineffective at equivalent concentrations; (e) *EP4* siRNA1 was selected for effective transient silencing of *EP4* expression in WT MCF-7; (f) transient silencing of *EP4* reduced sensitivity to TAM, only in the presence of PGE_2_. * denotes *P* < 0.05; ** denotes *P* < 0.002; *** denotes *P* < 0.005 normalised to respective vehicle controls

Since *HPGD* is responsible for the biological inactivation of prostaglandins including PGE_2_, we tested whether increased PGE_2_ signaling might explain TAMr. We first examined PGE_2_ levels in serum-free conditioned medium collected from MCF-7 and MCF-7 TAMr 2 cells. TAMr cells released higher amounts of PGE_2_ compared with parental MCF-7 cells and this could be reduced to levels approaching that of MCF-7 by *HPGD* overexpression in these cells (Figure 3a). Next, we tested the effect of adding exogenous PGE_2_ (1 µM) on TAM response in two different ERα-positive breast cancer cell lines, MCF-7 and T47D. PGE_2_ reduced sensitivity of both cell lines to TAM (Figure 3b). As PGE_2_-signalling is mediated by G-protein coupled receptors, we examined if the stimulatory EP2 or EP4 receptors might be responsible for mediating the effects of PGE_2_ on TAM sensitivity. We demonstrated that PGE_1_-OH (an EP4 agonist) was able to mimic the effects PGE_2_ in decreasing TAM sensitivity (Figure 3c; *P* < 0.015), albeit to a lesser extent than PGE_2_. However, butaprost (EP2 agonist) had no effect at equivalent concentrations (Figure 3d). This suggested that the EP4 receptor might be responsible for mediating the effect of PGE_2_ on TAM sensitivity. Partial silencing of *EP4* in WT MCF-7 cells was achieved using one siRNA (Figure 3e). *EP4* silencing using siRNA 1 inhibited PGE_2_ induced TAM resistance in these cells (Figure 3f).

### Loss of *HPGD* expression predicts worse outcomes in breast cancer

**Figure 4.**
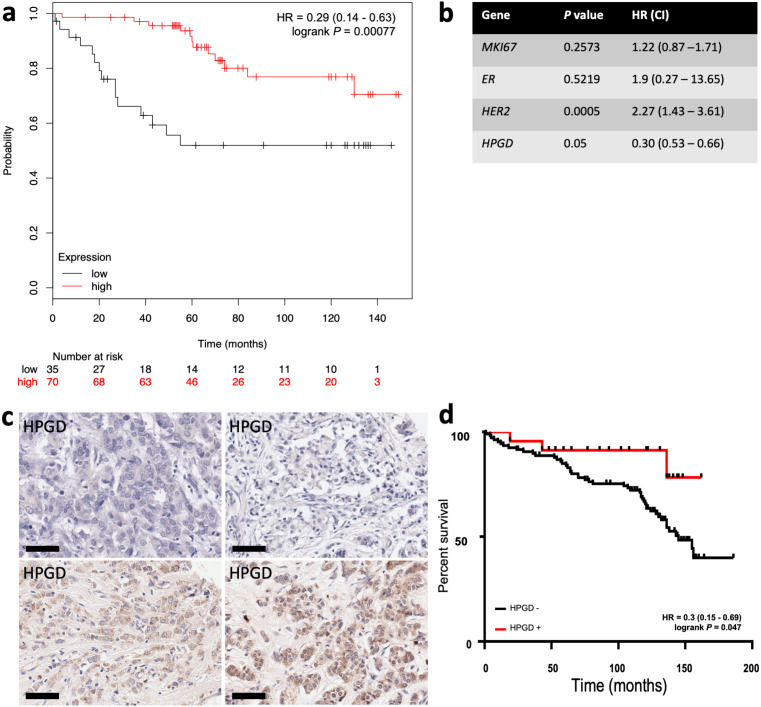
Kaplan-Meier survival analysis of the relationship between HPGD expression and breast cancer outcomes using *in silico* data mining and immunohistochemistry. Using KMplot, HPGD expression was categorised as ‘high’ (red) or ‘low’ (black), with the lower tertile expression used as cut-off. (a) Low *HPGD* expression is associated significantly reduced OS [HR 0.28 (0.13–0.59), *P* = 0.00041] in ERα breast tumours treated with TAM; (b) the association between *HPGD* expression and OS remained significant by multivariate analysis; (c) examples of HPGD staining (brown, DAB staining) in breast cancer tissue sections counterstained with haematoxylin (blue), the scale bar indicating 60 µm with some cases lacking any appreciable HPGD (top panels), and others showing weak to moderate staining (bottom panel); (d) a retrospective immunohistochemical analysis of HPGD expression in 130 breast cancers represented on TMAs showed HPGD-negative cases (black) had significantly worse OS [HR = 0.3 (0.15–0.69), *P* = 0.047] compared with HPGD-positive cases (red)

We analysed the relationship between HPGD expression and clinical outcome for breast cancer patients using the on-line resource Kmplot [30] and from our own cohort of breast cancer patient’s data in separate survival analyses. In the Kmplot cohort, we found that ER+ patients who received adjuvant TAM only and had ‘low’ expression of *HPGD*, had reduced OS by univariate analysis [hazard ratio (HR) = 0.28 (0.13–0.59), *P* = 0.0007] (Figure 4a). This remained significant on multivariate analysis (*P* = 0.001, Figure 4b). We then conducted a retrospective immunohistochemical study of HPGD expression in our own cohort of breast patients, who all received adjuvant TAM therapy and related HPGD expression to survival. As these TMAs have been used extensively in several other studies [24, 29, 35], the number of viable cores that could be reliably evaluated was only 144 out of 350 cases. The level of HPGD staining was generally weak to moderate (Figure 4c) in positive cases, however cases which completely lacked HPGD were associated with worse OS [HR = 0.3 (0.15–0.69), *P* = 0.047] (Figure 4d), in line with the *in silico* analysis (Figure 4a). The small number of TMA cases available precluded multivariate analysis.

### *In silico* identification of miRNA-*HPGD* target interactions

With growing evidence that *HPGD* is inactivated epigenetically, we sought to identify miRNAs that may post-transcriptionally modulate *HPGD* expression. We interrogated a global miRNA data set established in an independent TAM resistance model MCF-7:5C [33]. These TAMr cells were derived from WT MCF-7 cells (Table 1). *In silico* analysis revealed numerous miRNAs that were differentially expressed in TAMr MCF-7:5C cells compared with TAMs MCF-7:WS8 cells and that are predicted to interact with the three prime untranslated region (3′-UTR) of the *HPGD* transcripts (Table 2).

**Table 2.** *In silico* prediction of miRNAs differentially expressed in TAMr MCF-7:5C *vs.* TAMs MCF-7:WS8 that are predicted to interact with 3’UTR of major HPGD transcripts

**miRNA**	**Fold change**	**Ensembl id^a^**	**Binding probability^b^**	**Longest consecutive pairings^b^**
hsa-miR-432-5p	105.24	ENST00000504433	0.85	7
hsa-miR-382-5p	54.71	ENST00000296521, ENST00000542498	0.85	9
hsa-miR-487a-3p ^c^	42.78	ENST00000296522.6	-	-
hsa-miR-31-5p	28.67	ENST00000296522, ENST00000296521, ENST00000422112, ENST00000510901, ENST00000541923, ENST00000542498	1.0	14
hsa-miR-543	19.49	ENST00000296522, ENST00000296521, ENST00000422112, ENST00000541923, ENST00000542498/ENST00000510901	0.85/0.92	17
hsa-miR-493-3p	13.35	ENST00000296522, ENST00000296521, ENST00000422112, ENST00000541923, ENST00000542498/ENST00000510901	0.85/0.88	19
hsa-miR-376c-3p ^c^	10.65	ENST00000296522.6	-	-
hsa-miR-337-5p	6.46	ENST00000296522, ENST00000296521, ENST00000422112, ENST00000541923, ENST00000542498/ENST00000510901	0.85/0.92	7
hsa-miR-505-5p	6.32	ENST00000296522, ENST00000422112, ENST00000504433, ENST00000510901/ENST00000541923	1.0/0.85	6
hsa-miR-1972	5.64	ENST00000504433	0.92	7
hsa-miR-410-3p ^c^	4.00	ENST00000296522.6	-	-
hsa-miR-154-5p	3.39	ENST00000504433	0.85	5
hsa-miR-485-5p	3.08	ENST00000296522, ENST00000296521, ENST00000422112, ENST00000510901, ENST00000541923, ENST00000542498	1.0	7
hsa-miR-199a-3p^c^	2.91	ENST00000296522.6	-	-
hsa-miR-654-3p ^c^	2.89	ENST00000296522.6	-	-
hsa-miR-493-5p	2.68	ENST00000296522, ENST00000296521, ENST00000422112, ENST00000542498	0.85	10
hsa-miR-381-3p	2.66	ENST00000510901	0.85	8
hsa-miR-330-3p	2.54	ENST00000504433/ENST00000510901/ENST00000542498	0.92/0.85/1	9/7/7
hsa-miR-154-3p ^c^	2.35	ENST00000296522.6	-	-
hsa-miR-30a-3p	1.88	ENST00000504433	0.92/0.85	10/8
hsa-miR-4298	1.83	ENST00000296522, ENST00000296521, ENST00000422112, ENST00000510901, ENST00000541923, ENST00000542498	1.0	8
hsa-miR-3200-3p^c^	1.82	ENST00000296522.6	-	-
hsa-miR-106a-5p^c^	1.75	ENST00000296522.6	-	-
hsa-miR-20b-5p^c^	1.74	ENST00000296522.6	-	-
hsa-miR-425-5p^c^	1.71	ENST00000296522.6	-	-
hsa-miR-17-5p	1.69	ENST00000504433	0.85	10
hsa-miR-500a-5p	1.66	ENST00000296522, ENST00000296521, ENST00000422112, ENST00000541923, ENST00000542498, ENST00000510901	0.92	8
hsa-miR-1293	1.60	ENST00000296522, ENST00000296521, ENST00000422112, ENST00000541923, ENST00000542498, ENST00000510901	1.0	9
hsa-miR-20a-5p	1.56	ENST00000504433	0.92	10

Listed are miRNAs with fold change > 1.5; miRNAs underlined refer to the DLK-DIO3 locus on chromosome 14; miRWalk 3.0 [31] and TargetScan 7.2 [32] tools have been used to identify miRNA-target interactions; ^a^ major targeted transcripts are listed with respective Ensembl id; ^b^ binding probability and longest consecutive pairings are listed according to miRWalk 3.0 predictions; ^c^ additional miRNAs predicted only with TargetScan 7.2 tool (on most prevalent ENST00000296522.6 transcript)

**Figure 5.**
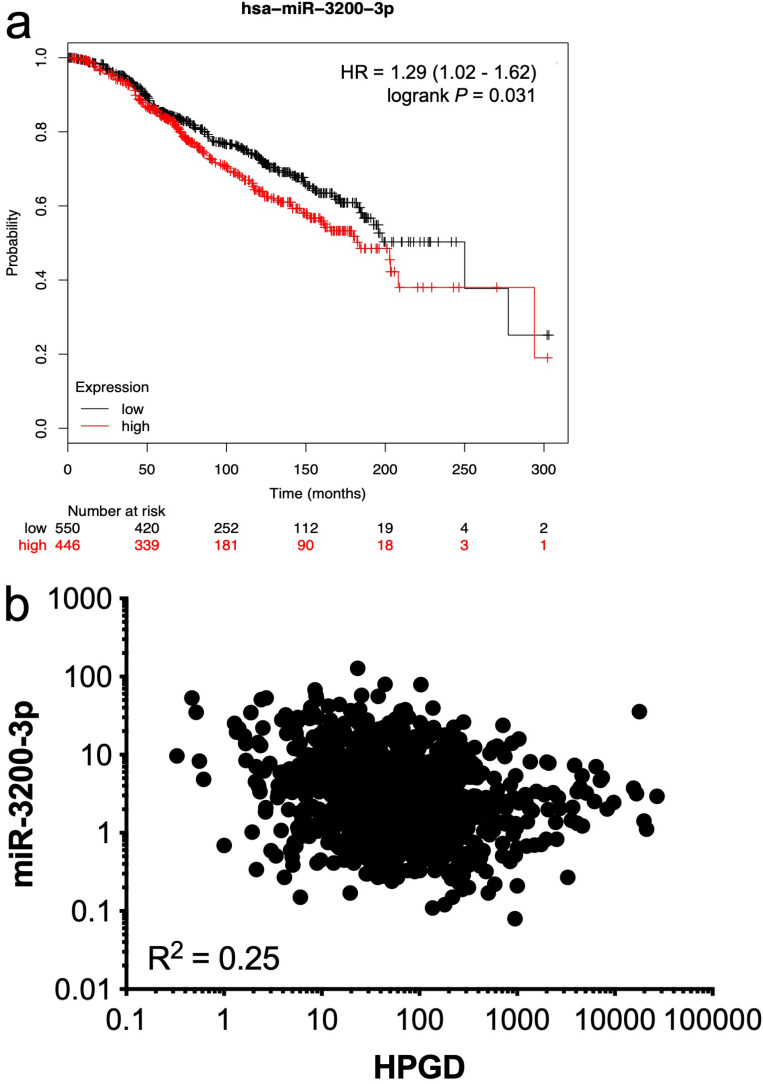
Relationship between *HPGD* and miR-3200-3p expression in breast cancer. Using KMplot, miR-3200-3p expression was categorized as high or low, with median expression used as cut-off. Kaplan-Meier survival analysis demonstrated a statistically significant effect of miR-3200-3p on the OS of ERα-positive breast cancer patients treated with any endocrine therapy (a); correlation analysis of matched gene expression levels of *HPGD* and miR-3200-3p in 987 breast cancer cases obtained from the TCGA revealed a weak negative correlation [R^2^ = 0.25; (b)]

We then tested the association between each of the listed miRNAs and *HPGD* mRNA levels in 987 breast cancer samples from the TCGA database using OncoLnc [34] where both miRNA and mRNA expression data were available. Only one miRNA, namely miR-3200-3p, had an inverse relationship between its expression levels and *HPGD* mRNA levels in this dataset (Spearman, ρ = –0.25, *P* < 0.0001). We used the Kmplot online resource [30] to analyse the association between miR-3200-3p expression and OS in ER+ breast cancer patients treated with any endocrine therapy, irrespective of grade, molecular subtype, and lymph node status. High miR-3200-3p expression was associated with significantly reduced OS (Figure 5a) and a weak negative correlation (R^2^ = 0.25) was observed between miR-3200-3p and *HPGD* (Figure 5b) using data from TCGA analysed using the OncoLnc platform [34].

## Discussion

We have demonstrated a downregulation of HPGD expression in TAM-resistant breast cancer both *in vitro* in multiple series of TAMs and TAMr isogenic cell line pairs and in clinical samples using retrospective immunohistochemistry studies and *in silico* approaches. We have also shown that TAM resistance is associated with PGE_2_ signalling via the EP4 receptor *in vitro*.

A key role of *HPGD* is to inactivate PGE_2_. Having confirmed its downregulation in TAMr cells using various experimental approaches and in different cell lineages, we showed that stable transfection of human *HPGD* into MCF-7 TAMr 2MCF-7 cells restored HPGD enzyme activity, at the same time significantly sensitising these cells to TAM, and suggesting a functional role of *HPGD* downregulation in acquired TAMr. Our hypothesis that *HPGD* mediated its effects via this axis was strengthened by the observation that treatment of 2 different ER+ TAM-sensitive cell lines with exogenous PGE_2_ increased the resistance of both cell lines to the growth-inhibitory effects of TAM.

PGE_2_ exerts its effects through binding to cell surface G-protein coupled EP receptors. Four subtypes have been identified, EP1, -2, -3 and -4, with each coupled to different intracellular signalling pathways. All are stimulatory except for EP3, which exerts inhibitory effects. We used a pharmacological approach to target EP2 and EP4, treating parental MCF-7 and T47D cells with PGE_1_-OH (EP4 receptor agonist) or with butaprost (EP2 receptor agonist). PGE_1_-OH was able to mimic the effects of PGE_2_ (albeit to a lesser extent), whereas butaprost had no effect at equivalent concentrations. We suggest that the EP4 receptor is responsible for mediating the TAMr effect of PGE_2_. While our knockdown experiment implied a role for EP4, we were only able to achieve partial knockdown with a single siRNA, which limits the conclusions from our data. Consistent with our data, a study has shown that EP4 gene expression was upregulated in TAMr MCF-7 cells, also generated from long term oestrogen deprivation, and in patients resistant to aromatase inhibitor therapy. Furthermore, the exposure to two separate EP4-specific antagonists, GW627368X and ONO-AE3-208, decreased estrogen-independent cell growth in LTED MCF-7 cells [36]. These authors proposed a molecular mechanism by which EP4 signalling may drive ligand-independent ERα activation by driving the binding of the coactivator-associated arginine methyltransferase 1 (CARM1), an ERα co-factor, to the receptor, leading to its activated status [36].

There is evidence that *HPGD* is post-transcriptionally regulated via miRNAs, with miR-21 identified as targeting *HPGD* in oral tongue squamous cell carcinoma [37]. Silencing of miR-21 expression in human breast cancer cell lines has been shown to increase TAM sensitivity [38]. miR-21 was found to be overexpressed in TAMr cell line compared to TAMs cell lines [39], which may contribute to *HPGD* downregulation in TAM resistant breast cancer. However, miR-21 was not identified as differentially expressed in TAMr MCF-7:5C cells compared with TAMs MCF-7:WS8 cells. We identified several differentially expressed miRNAs predicted to interact with the 3’UTR of *HPGD* transcripts. Of these, miR-3200-3p expression was found to be inversely correlated with *HPGD* mRNA level, suggesting a possible role of miR-3200-3p in the regulation of HPGD expression. Using an *in silico* approach we found that increased miR-3200-3p levels were significantly associated with decreased OS in ER positive breast cancer patients treated with endocrine therapy. There are no data available thus far establishing a direct interaction between miR-3200-3p and *HPGD* mRNA, however correlation between *HPGD* and miR-3200-3p in breast cancer cases in the TCGA database using the OncoLnc platform [34] revealed a weak negative correlation. A key next step would be to validate that the 3′-UTR of *HPGD* is a target of miR-3200-3p in a luciferase reporter assay *in vitro*, with *in vivo* validation also warranted. Interestingly, while this work was under review, miR-3200-3p was identified using data from TCGA as a so-called miRNA master regulator (MMR) in breast cancer and was one of 61 MMRs which displayed high oncogenic activity [40].

The immunohistochemistry on human breast cancer samples and the *in silico* analysis demonstrated that reduced expression of HPGD was associated with worse patient outcomes, consistent with a study demonstrating that low levels of HPGD predicted reduced recurrence free survival and OS in a cohort of breast cancer patients, split into normal weight, overweight and obese groups, in which few received endocrine therapy (15%) [41]. In our study, all cases received adjuvant TAM, with HPGD downregulation associated with reduced response to endocrine treatment and worse clinical outcomes.

Some studies of *HPGD* expression in breast tissues have demonstrated higher expression in normal breast tissue than breast cancer [19], supporting a tumour suppressive role [17, 42]. However, this is an inconsistent finding. One study showed that high, rather than low, *HPGD* expression was significantly associated with poor outcome in triple negative breast cancer [18]. However, tumours lacking expression of either ERα, PR and HER2 are recognised to have worse prognosis. Our cohort was predominantly ER-positive (77%) and the *in silico* analysis was restricted to ER-positive cases only. Such context-dependency has been supported through a data mining approach where *HPGD* expression was found to be differentially expressed across the various molecular subtypes of breast cancer [19]. However, in this study [19], *HPGD* expression was significantly decreased in triple negative breast cancer compared to other molecular subtypes.

Data extrapolated from clinical trials is beginning to indicate that the risk of breast cancer relapse is reduced by extending time on endocrine therapy from 5 to 10 years [43]. While this is good for patient outcome, there will likely be a lag period before these benefits are seen in the clinic. Understanding the mechanisms which underline endocrine therapy resistance remains an important biological question. This study demonstrated that HPGD downregulation in ER-positive breast cancer is associated with reduced response to adjuvant TAM therapy via PGE_2_-EP4 signalling and decreased patient survival. These data offer a potential target to develop combination therapies that may overcome TAM acquired resistance.

## Abbreviations

3′-UTR: three prime untranslated region
CARM1: coactivator-associated arginine methyltransferase
COX: cyclooxygenase
E2: 17β-oestradiol
EP4: prostaglandin E_2_ receptor 4
ERα: oestrogen receptor alpha
FCS: foetal calf serum
HPGD: 15-hydroxyprostaglandin dehydrogenase
LTED: long-term estrogen deprivation
MMR: miRNA master regulator
MTT: 3-(4,5-dimethylthiazol-2-yl)-2,5-diphenyltetrazolium bromide
OH: alcohol
OS: overall survival
PGE_2_: prostaglandin E_2_
PR: progesterone receptor
qRT-PCR: real-time quantitative polymerase chain reaction
RT-PCR: real-time polymerase chain reaction
SD: standard deviation
siRNA: small-interfering RNA
TAM: Tamoxifen
TAMr: tamoxifen-resistant
TAMs: tamoxifen-sensitive
TCGA: the cancer genome atlas
TMA: tissue microarray
WT: wild type

## Declarations

### Availability of data and materials

Part of the results described is based upon data generated by the TCGA Research Network (https://www.cancer.gov/about-nci/organization/ccg/research/structural-genomics/tcga), the online resources of KMplot (https://kmplot.com/analysis/) and OncoLnc (http://www.oncolnc.org/).

### Copyright

© The Author(s) 2020.

## References

[1] Jordan VC. Tamoxifen: catalyst for the change to targeted therapy. Eur J Cancer. 2008;44:30–8. 10.1016/j.ejca.2007.11.002 18068350 PMC2566958

[2] Early Breast Cancer Trialists’ Collaborative Group (EBCTCG). Aromatase inhibitors *versus* tamoxifen in early breast cancer: patient-level meta-analysis of the randomised trials. Lancet. 2015;386:1341–52. 10.1016/S0140-6736(15)61074-1 26211827

[3] Mathew A Davidson NE. Adjuvant endocrine therapy for premenopausal women with hormone-responsive breast cancer. Breast. 2015;24 Suppl 2:120–5. 10.1016/j.breast.2015.07.027 26255743

[4] Madsen MW Reiter BE Lykkesfeldt AE. Differential expression of estrogen receptor mRNA splice variants in the tamoxifen resistant human breast cancer cell line, MCF-7/TAMR-1 compared to the parental MCF-7 cell line. Mol Cell Endocrinol. 1995;109:197–207. 10.1016/0303-7207(95)03503-y 7664983

[5] Coopman P Garcia M Brunner N Derocq D Clarke R Rochefort H. Anti-proliferative and anti-estrogenic effects of ICI 164,384 and ICI 182,780 in 4-OH-tamoxifen-resistant human breast-cancer cells. Int J Cancer. 1994;56:295–300. 10.1002/ijc.2910560225 8314314

[6] Jordan NJ Gee JM Barrow D Wakeling AE Nicholson RI. Increased constitutive activity of PKB/Akt in tamoxifen resistant breast cancer MCF-7 cells. Breast Cancer Res Treat. 2004;87:167–80. 10.1023/B:BREA.0000041623.21338.47 15377841

[7] Brunner N Frandsen TL Holst-Hansen C Bei M Thompson EW Wakeling AE MCF7/LCC2: a 4-hydroxytamoxifen resistant human breast cancer variant that retains sensitivity to the steroidal antiestrogen ICI 182,780. Cancer Res. 1993;53:3229–32. 8324732

[8] Brunner N Boysen B Jirus S Skaar TC Holst-Hansen C Lippman J MCF7/LCC9: an antiestrogen-resistant MCF-7 variant in which acquired resistance to the steroidal antiestrogen ICI 182,780 confers an early cross-resistance to the nonsteroidal antiestrogen tamoxifen. Cancer Res. 1997;57:3486–93. 9270017

[9] Limer JL Parkes AT Speirs V. Differential response to phytoestrogens in endocrine sensitive and resistant breast cancer cells *in vitro*. Int J Cancer. 2006;119:515–21. 10.1002/ijc.21863 16506217

[10] Scott DJ Parkes AT Ponchel F Cummings M Poola I Speirs V. Changes in expression of steroid receptors, their downstream target genes and their associated co-regulators during the sequential acquisition of tamoxifen resistance *in vitro*. Int J Oncol. 2007;31:557–65. 10.3892/ijo.31.3.557 17671682

[11] Backlund MG Mann JR Holla VR Buchanan FG Tai HH Musiek ES 15-Hydroxyprostaglandin dehydrogenase is down-regulated in colorectal cancer. J Biol Chem. 2005;280:3217–23. 10.1074/jbc.M411221200 15542609 PMC1847633

[12] Yan M Rerko RM Platzer P Dawson D Willis J Tong M 15-Hydroxyprostaglandin dehydrogenase, a COX-2 oncogene antagonist, is a TGF-beta-induced suppressor of human gastrointestinal cancers. Proc Natl Acad Sci U S A. 2004;101:17468–73. 10.1073/pnas.0406142101 15574495 PMC536023

[13] Ding Y Tong M Liu S Moscow JA Tai HH. NAD+-linked 15-hydroxyprostaglandin dehydrogenase (15-PGDH) behaves as a tumor suppressor in lung cancer. Carcinogenesis. 2005;26:65–72. 10.1093/carcin/bgh277 15358636

[14] Tseng-Rogenski S Gee J Ignatoski KW Kunju LP Bucheit A Kintner HJ Loss of 15-hydroxyprostaglandin dehydrogenase expression contributes to bladder cancer progression. Am J Pathol. 2010;176:1462–8. 10.2353/ajpath.2010.090875 20093479 PMC2832165

[15] Thiel A Ganesan A Mrena J Junnila S Nykanen A Hemmes A 15-hydroxyprostaglandin dehydrogenase is down-regulated in gastric cancer. Clin Cancer Res. 2009;15:4572–80. 10.1158/1078-0432.CCR-08-2518 19584167

[16] Cummings M Massey KA Mappa G Wilkinson N Hutson R Munot S Integrated eicosanoid lipidomics and gene expression reveal decreased prostaglandin catabolism and increased 5-lipoxygenase expression in aggressive subtypes of endometrial cancer. J Pathol. 2019;247:21–34. 10.1002/path.5160 30168128

[17] Wolf I O’Kelly J Rubinek T Tong M Nguyen A Lin BT 15-hydroxyprostaglandin dehydrogenase is a tumor suppressor of human breast cancer. Cancer Res. 2006;66:7818–23. 10.1158/0008-5472.CAN-05-4368 16885386

[18] Lehtinen L Vainio P Wikman H Reemts J Hilvo M Issa R 15-Hydroxyprostaglandin dehydrogenase associates with poor prognosis in breast cancer, induces epithelial-mesenchymal transition, and promotes cell migration in cultured breast cancer cells. J Pathol. 2012;226:674–86. 10.1002/path.3956 22072156

[19] Kochel TJ Goloubeva OG Fulton AM. Upregulation of cyclooxygenase-2/prostaglandin E2 (COX-2/PGE2) pathway member multiple drug resistance-associated protein 4 (MRP4) and downregulation of prostaglandin transporter (PGT) and 15-prostaglandin dehydrogenase (15-PGDH) in triple-negative breast cancer. Breast Cancer (Auckl). 2016;10:61–70. 10.4137/BCBCR.S38529 27257388 PMC4881873

[20] Celis JE Gromova I Gromov P Moreira JM Cabezón T Friis E Molecular pathology of breast apocrine carcinomas: a protein expression signature specific for benign apocrine metaplasia. FEBS Lett. 2006;580:2935–44. 10.1016/j.febslet.2006.03.080 16631754

[21] Bhar A Haubrock M Mukhopadhyay A Maulik U Bandyopadhyay S Wingender E. Coexpression and coregulation analysis of time-series gene expression data in estrogen-induced breast cancer cell. Algorithms Mol Biol. 2013;8:9. 10.1186/1748-7188-8-9 23521829 PMC3827943

[22] Brunner N Boulay V Fojo A Freter CE Lippman ME Clarke R. Acquisition of hormone-independent growth in MCF-7 cells is accompanied by increased expression of estrogen-regulated genes but without detectable DNA amplifications. Cancer Res. 1993;53:283–90. 8380254

[23] Jiang SY Wolf DM Yingling JM Chang C Jordan VC. An estrogen receptor positive MCF-7 clone that is resistant to antiestrogens and estradiol. Mol Cell Endocrinol. 1992;90:77–86. 10.1016/0303-7207(92)90104-e 1301400

[24] Maraqa L Cummings M Peter MB Shaaban AM Horgan K Hanby AM Carcinoembryonic antigen cell adhesion molecule 6 predicts breast cancer recurrence following adjuvant tamoxifen. Clin Cancer Res. 2008;14:405–11. 10.1158/1078-0432.CCR-07-1363 18223215

[25] Ponchel F Toomes C Bransfield K Leong FT Douglas SH Field SL Real-time PCR based on SYBR-Green I fluorescence: an alternative to the TaqMan assay for a relative quantification of gene rearrangements, gene amplifications and micro gene deletions. BMC Biotechnol. 2003;3:18. 10.1186/1472-6750-3-18 14552656 PMC270040

[26] Young AL Chalmers CR Hawcroft G Perry SL Treanor D Toogood GJ Regional differences in prostaglandin E_2_ metabolism in human colorectal cancer liver metastases. BMC Cancer. 2013;13:92. 10.1186/1471-2407-13-92 23442768 PMC3598740

[27] Tai HH. Enzymatic synthesis of (15s)-[15-3h]prostaglandins and their use in the development of a simple and sensitive assay for 15-hydroxyprostaglandin dehydrogenase. Biochemistry. 1976;15:4586–92. 10.1021/bi00666a007 184819

[28] Al-Nakhle H Burns PA Cummings M Hanby AM Hughes TA Satheesha S Estrogen receptor β1 expression is regulated by miR-92 in breast cancer. Cancer Res. 2010;70:4778–84. 10.1158/0008-5472.CAN-09-4104 20484043 PMC2883739

[29] Wong PP Yeoh CC Ahmad AS Chelala C Gillett C Speirs V Identification of MAGEA antigens as causal players in the development of tamoxifen-resistant breast cancer. Oncogene. 2014;33:4579–88. 10.1038/onc.2014.45 24662835 PMC4162461

[30] Gyorffy B Lanczky A Eklund AC Denkert C Budczies J Li Q An online survival analysis tool to rapidly assess the effect of 22,277 genes on breast cancer prognosis using microarray data of 1,809 patients. Breast Cancer Res Treat. 2010;123:725–31. 10.1007/s10549-009-0674-9 20020197

[31] Dweep H Sticht C Pandey P Gretz N. miRWalk--database: prediction of possible miRNA binding sites by “walking” the genes of three genomes. J Biomed Inform. 2011;44:839–47. 10.1016/j.jbi.2011.05.002 21605702

[32] Agarwal V Bell GW Nam JW Bartel DP. Predicting effective microRNA target sites in mammalian mRNAs. Elife. 2015;4:e05005. 10.7554/eLife.05005.001 PMC4532895 26267216

[33] Hoppe R Fan P Büttner F Winter S Tyagi AK Cunliffe H Profiles of miRNAs matched to biology in aromatase inhibitor resistant breast cancer. Oncotarget. 2016;7:71235–54. 10.18632/oncotarget.12103 27659519 PMC5342075

[34] Anaya J. OncoLnc: linking TCGA survival data to mRNAs, miRNAs, and lncRNAs. Peerj Computer Sci. 2016;2:e67. 10.7717/peerj-cs.67

[35] Hamilton-Burke W Coleman L Cummings M Green CA Holliday DL Horgan K Phosphorylation of estrogen receptor beta at serine 105 is associated with good prognosis in breast cancer. Am J Pathol. 2010;177:1079–86. 10.2353/ajpath.2010.090886 20696772 PMC2928942

[36] Hiken JF McDonald JI Decker KF Sanchez C Hoog J VanderKraats ND Epigenetic activation of the prostaglandin receptor EP4 promotes resistance to endocrine therapy for breast cancer. Oncogene. 2017;36:2319–27. 10.1038/onc.2016.397 27869171 PMC5398938

[37] He Q Chen Z Dong Q Zhang L Chen D Patel A MicroRNA-21 regulates prostaglandin E2 signaling pathway by targeting 15-hydroxyprostaglandin dehydrogenase in tongue squamous cell carcinoma. BMC Cancer. 2016;16:685. 10.1186/s12885-016-2716-0 27561985 PMC5000501

[38] Yu X Li R Shi W Jiang T Wang Y Li C Silencing of MicroRNA-21 confers the sensitivity to tamoxifen and fulvestrant by enhancing autophagic cell death through inhibition of the PI3K-AKT-mTOR pathway in breast cancer cells. Biomed Pharmacother. 2016;77:37–44. 10.1016/j.biopha.2015.11.005 26796263

[39] Ye P Fang C Zeng H Shi Y Pan ZY An NR Differential microRNA expression profiles in tamoxifen-resistant human breast cancer cell lines induced by two methods. Oncol Lett. 2018;15:3532–9. 10.3892/ol.2018.7768 29467872 PMC5796357

[40] Martinez-Gutierrez AD Cantú de León D Millan-Catalan O Coronel-Hernandez J Campos-Parra AD Porras-Reyes F Identification of miRNA master regulators in breast cancer. Cells. 2020;9:1610. 10.3390/cells9071610 PMC7407970 32635183

[41] Wu R Liu T Yang P Liu X Liu F Wang Y Association of 15-hydroxyprostaglandin dehydrogenate and poor prognosis of obese breast cancer patients. Oncotarget. 2017;8:22842–53. 10.18632/oncotarget.15280 28206964 PMC5410267

[42] Tai HH. Prostaglandin catabolic enzymes as tumor suppressors. Cancer Metastasis Rev. 2011;30:409–17. 10.1007/s10555-011-9314-z 22020925

[43] Pan H Gray R Braybrooke J Davies C Taylor C McGale P 20-year risks of breast-cancer recurrence after stopping endocrine therapy at 5 years. N Engl J Med. 2017;377:1836–46. 10.1056/NEJMoa1701830 29117498 PMC5734609

